# Graphene quantum dots (GQD) and edge-functionalized GQDs as hole transport materials in perovskite solar cells for producing renewable energy: a DFT and TD-DFT study

**DOI:** 10.1039/d3ra05438a

**Published:** 2023-10-04

**Authors:** Anjan Kumar, M. I. Sayyed, Diego Punina, Eugenia Naranjo, Edwin Jácome, Maha Khalid Abdulameer, Hamza Jasim Albazoni, Zahra Shariatinia

**Affiliations:** a Department of Electronics and Communication Engineering, GLA University Mathura-281406 India; b Department of Physics, Faculty of Science, Isra University Amman 11622 Jordan; c Department of Nuclear Medicine Research, Institute for Research and Medical Consultations (IRMC), Imam Abdulrahman bin Faisal University (IAU) PO Box 1982 Dammam 31441 Saudi Arabia; d Facultad de Ciencias de la Ingeniería, Carrera Ingeniería Mecánica, Universidad Técnica Estatal de Quevedo (UTEQ) Quevedo Ecuador; e Facultad Mecánica, Escuela Superior Politécnica de Chimborazo (ESPOCH) Riobamba 060155 Ecuador; f Facultad de Mecánica, Escuela Superior Politécnica de Chimborazo (ESPOCH) Riobamba 060155 Ecuador; g Department of Radiology & Sonar Techniques, Al-Noor University College Nineveh Iraq maha.khalid@alnoor.edu.iq; h College of Nursing, National University of Science and Technology Dhi Qar Iraq; i Department of Chemistry, Tehran Polytechnic, Amirkabir University of Technology P.O. Box: 15875-4413 Tehran Iran

## Abstract

This study investigated the potential suitability of graphene quantum dots (GQD) and certain edge-functionalized GQDs (GQD-3Xs) as hole transport materials (HTMs) in perovskite solar cells (PSCs). The criteria for appropriate HTMs were evaluated, including solubility, hole mobility, light harvesting efficiency (LHE), exciton binding energy (*E*_b_), hole reorganization energy (*λ*_h_), hole mobility, and HTM performance. It was found that several of the compounds had higher hole mobility than Spiro-OMeTAD, a commonly used HTM in PSCs. The open circuit voltage and fill factor of the suitable GQD and GQD-3Xs were found to be within appropriate ranges for HTM performance in MAPbI_3_ PSCs. GQD-COOH and GQD-COOCH_3_ were identified as the most suitable HTMs due to their high solubility, small *λ*_h_, and appropriate performance.

## Abstract 2

This study investigated the potential suitability of graphene quantum dots (GQD) and certain edge-functionalized GQDs (GQD-3Xs) as hole transport materials (HTMs) in perovskite solar cells (PSCs).

## Graphical abstract

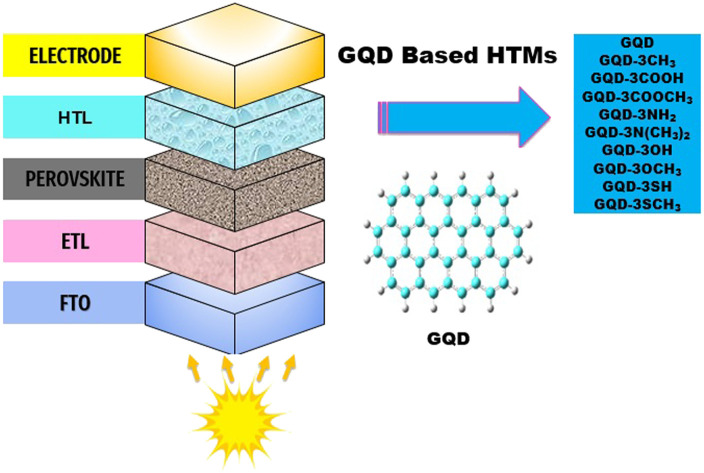

## Introduction

Hole transport materials (HTM) are of utmost importance in the optimization of perovskite solar cells (PSCs), which have garnered considerable interest from both scientists and industrial sectors owing to their remarkable characteristics like high efficiency, cost-effectiveness, and facile integration *via* roll-to-roll fabrication techniques.^1–3^ The PSC has emerged as a transformative technology in the realm of photovoltaics, exhibiting a significant improvement in efficiency from 3.7% to 25.8% within a very short timeframe. This remarkable progress positions PSCs as a strong contender to conventional silicon solar cells.^4,5^ PSCs often have a light-absorbing level positioned between the HTM and the electron transport material (ETM). The primary function of the HTM is to assist in the extraction of holes while impeding charge recombination at the interface between the HTM and the perovskite layer.^6^ In order to be deemed appropriate for use in PSCs, HTMs must possess significant attributes like extraordinary hole transport capacity and conductivity, high mobility, a HOMO level that is well-aligned with the valence level of the perovskite material, favorable solubility to facilitate processability and cost-effectiveness.^7,8^ The effectiveness of a PSC device is significantly affected by the choice of HTMs since they fulfil many crucial functions. There are two primary functions of HTMs. Firstly, they serve to alter the energy barrier height that exists between the active layer and the electrode. Secondly, they create a specialized contact for the carriers. Furthermore, it is important to note that HTMs play a crucial role in providing a protective barrier, effectively safeguarding the active layer from any physical or chemical interactions that may arise between the electrode and the active layer. In addition, these entities play a crucial role in enhancing the process of charge transmission and collecting, hence contributing to the overall efficiency of the device. Finally, it should be noted that the use of HTMs has a remarkable role in enhancing the stability of both the active layer and the electrode. This aspect holds immense importance as it directly influences the overall stability of the device.^4,8^

Spiro-OMeTAD, scientifically referred to as 2,2′,7,7′-tetrakis-(*N*,*N*-di-*p*-methoxyphenylamine), the compound known as 9,9′-spirobifluorene has been more favored as a HTM in the advancement of PSCs. This is primarily attributed to its stable amorphous structure, capacity to generate thin films, and favorable electrical conductivity subsequent to doping.^9–13^ However, the high cost involved in its synthesis has prompted researchers to explore alternative materials.^14^ Fortunately, several simple and inexpensive alternatives for HTMs have been successfully developed, including fluorene,^15^ phenylamines,^16^ carbazole,^17,18^ furan,^19^ azine,^20^ silolothiophene,^21^ and azomethine.^22^ Carbon quantum dots (CQDs) have garnered significant interest in recent years owing to their distinctive characteristics and prospective uses as luminous nanomaterials.^23–28^ The first discovery of these entities occurred in 2004 by Xu *et al.* Subsequent investigations have been conducted to gain insights into their synthesis, characterisation, and prospective applications.^27,29^ CQDs are renowned for their remarkable stability and conductivity, little toxicity, and ecologically benign characteristics, making them very appealing for a different type of applications.^27,28^

Graphene quantum dots (GQDs) have received most attention in contemporary discourse as a variant of CQDs. The GQDs are often characterized by their small dimensions, generally measuring less than 10 nanometers, and are mostly composed of carbon atoms.^30–32^ However, it is possible to functionalize them in order to alter their features, such as adjusting the band gap, to cater to diverse applications. Carbon quantum dots (C-dots) are carbon atoms at the periphery of GQDs that have been fully saturated with hydrogen atoms.^33^ C-dots have been the subject of substantial research due to their potential uses, notably in the field of photocatalysis. Numerous studies have shown the ability of carbon dots (C-dots) to function as exceptionally proficient photocatalysts, exhibiting remarkable catalytic efficacy. Furthermore, it has been shown that the boundaries of carbon dots (C-dots) possess the ability to impact the process of light absorption, hence emphasising its promising prospects in the field of photocatalysis.^34–36^

In recent times, there has been a growing attention in the use of CQDs, with a particular focus on GQDs, as highly efficient HTMs in the PSC. This interest stems from the remarkable attributes of CQDs, including their stability, non-toxic nature, cost-effectiveness, and environmentally friendly properties.^37–39^ A technique for producing nitrogen-functionalized graphene quantum dots (NGQDs) with adjustable optical properties was developed by Testsuka *et al.* Incorporating NGQD layers into optoelectronic devices, such as perovskite solar cells and hybrid phototransistors, significantly improved their performance.^37^ Matta *et al.* employed density functional theory to examine a range of pristine and functionalized hexagonal GQDs and reported that GQDs functionalized with –OH and –COOH were effective for hole transport in MAPbI_3_ as a PSCs.^38^ Furthermore, Sorli *et al.* investigated the potential of 360 different coronene derivatives as small GQDs for hole transport, using both theoretical and experimental methods.^39^ The present study aims to utilize DFT to examine the impact of the edge substitutions on the efficiency of GQDs as HTMs in perovskite solar cells.

This study investigates the potential of Graphene Quantum Dots (GQD) and a particular kind of edge-functionalized GQDs, referred to as GQD-3Xs, as Hole Transport Materials (HTMs) with prospective applications in PSCs. The careful choice of suitable heterojunction materials (HTMs) plays a pivotal role in optimizing the overall performance of PSCs. In order to assess the appropriateness of these materials, a thorough set of criteria is used, including solubility, hole mobility, light harvesting efficiency (LHE), exciton binding energy (*E*_b_), hole reorganization energy (*λ*_h_), hole mobility, and hole transport material (HTM) performance. The method used in our study encompasses a series of methodical inquiries aimed at comprehensively characterizing the material characteristics and evaluating the performance of Graphene Quantum Dots (GQDs) and Triple-Doped Graphene Quantum Dots (GQD-3Xs) inside the framework of PSCs.

## Computational details

The Berny optimization algorithm^40^ was used to optimize the GQD and 9 functionalized GQDs. During the optimization process, we selected maximum force, RMS force, maximum displacement, and RMS displacement thresholds of 4.5 × 10^−4^ Hartree/Bohr, 3.0 × 10^−4^, 1.8 × 10^−3^ Bohr, and 1.2 × 10^−3^, respectively. The influence of dichloromethane solvent were studied by employing conductor-like polarizable continuum model (C-PCM) method.^41^ Imaginary frequencies were not found in the frequency calculations performed on pristine and functionalized GQDs. The UV-visible (UV-Vis) spectra were obtained using time-dependent density functional theory (TD-DFT) calculations.^42^ Natural bond orbitals analysis^43^ accompanied with Chemcraft 1.7 (ref. 44) were employed to study and draw molecular orbitals of pristine and functionalized GQDs.

Eqn (1) and (2) were used to calculate the light harvesting efficiency (LHE) and exciton binding energy (*E*_b_) of molecules considered GQDs.^45–47^1
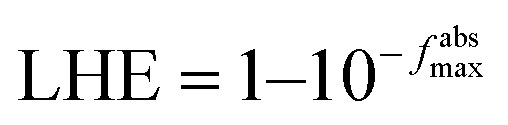

2 *E*_b_ = *E*_g_ − *E*_opt_

where, *f*^abs^_max_ is the oscillator strengths for the maximum absorption peak. *E*_g_ is defined as the gap energy between the HOMO and LUMO whereas *E*_opt_ is optical bandgap which is the gap energy between electronic ground state and first excited state.

All geometries were optimized using Grimme's dispersion corrected^48^ B3LYP hybrid functional (B3LYP-D3) with 6-31G** basis sets through Gaussian 09 quantum package.^49^ Also, B3LYP-D3 along with def2svp basis sets^50^ were employed for NBO and TD-DFT calculations. B3LYP-D3/def2svp level of theory has been proved to simulate the electronic structures of CQDs and GQDs with reliable accuracy.^39^

## Results and discussion

### Geometry, solubility, and dipole moment

**Fig. 1:**
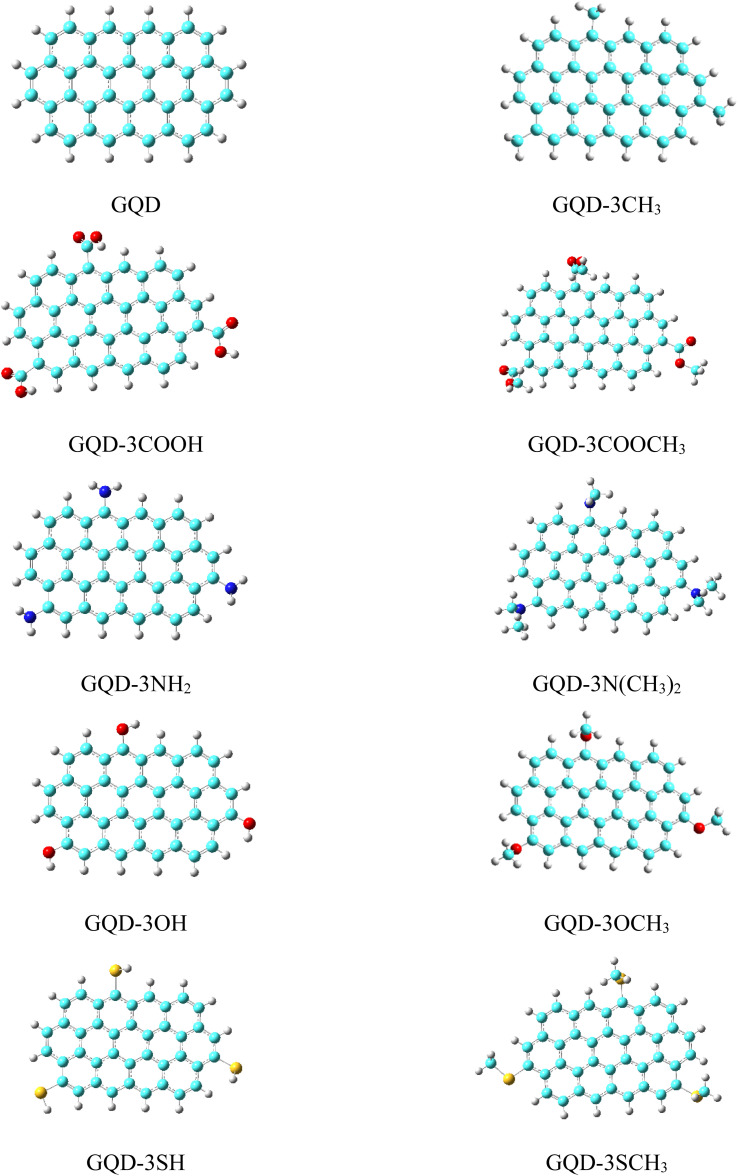
The optimized geometries of the GQD and GQD-3Xs.

The optimized geometries of the considered pristine and edge functionalized graphene quantum dots (GQD and GQD-3X, X = CH_3_, COOH, COOCH_3_, NH_2_, NMe_2_, OH, OMe, SH, and SMe) in gas phase have been depicted in Fig. 1. In the development of organic electronic devices, solubility is a critical characteristic of HTMs. This term describes the capacity of a substance to dissolve in a specific solvent and form a homogenous solution. The significance of solubility lies in its impact on the ability to create a thin film of the material, which is a crucial element for the efficient performance of electronic devices.^51^ The formula Δ*E*_solvation_ = *E*_solution_ − *E*_gas_ was utilized to obtain the solvation energies of HTMs, where *E*_solution_ and *E*_gas_, respectively, represent the molecular energies in the solvent and gas phase.^47^ The solvation energies of the GQD and GQD-3Xs in dichloromethane solvent have been imported in Table 1. The results indicate that the HTMs with the highest solvation energies are GQD-3COOH, GQD-3COOCH_3_, and GQD-3NH_2_, with values of −14.45, −12.92, and −11.68 kcal mol^−1^, respectively. These findings indicate that GQD-3COOH, GQD-3COOCH_3_, and GQD-3NH_2_ possess the greatest solubility among the HTMs considered. This phenomenon can be attributed to the strong hydrogen bonding ability of the –COOH, –COOCH_3_, and –NH_2_ substituents with dichloromethane.

**Table 1:** Solvation energy (Δ*E*_solvation_), dipole momentum (*D*), and polarizability (*α*) of the GQD and GQD-3Xs

	Δ*E*_solvation_ (kcal mol^−1^)	*D* (gas phase) (Debye)	*D* (solvation) (Debye)	*α* (a.u.)
GQD	−4.70	0.00	0.00	832.03
GQD-3CH_3_	−4.88	0.13	0.28	900.55
GQD-3COOH	−14.43	6.20	9.16	935.27
GQD-3COOCH_3_	−12.92	6.78	9.05	976.71
GQD-3NH_2_	−11.68	3.46	4.51	919.62
GQD-3N(CH_3_)_2_	−5.51	1.26	1.87	1005.93
GQD-3OH	−9.65	1.67	2.22	867.20
GQD-3OCH_3_	−6.74	3.45	4.44	920.59
GQD-3SH	−6.72	1.78	2.50	968.79
GQD-3SCH_3_	−6.51	3.43	4.38	1022.86

Table 1 also reports dipole moments of GQD and GQD-3Xs in gas phase and dichloromethane solvent. An inverse relationship exists between dipole moment and electron-carrier mobility. A high dipole moment indicates a low in electron-carrier mobility, while simultaneously improving the mobility of hole-carriers,^52^ improving organic electronic devices performance. GQD is a nonpolar compound due to its symmetric structure, which results in a lack of dipole moment. However, the introduction of edge functionalization can break its symmetry and enhance its dipole moment and polarity. Among the GQD-3Xs considered, GQD-3COOH and GQD-3COOCH_3_ have the highest polarity, with dipole moments of 6.20 and 6.78 *D* in gas phase, respectively. Furthermore, dissolving GQD-3Xs in dichloromethane can increase their polarities.

Other factor which plays an significant role in the transport of charge carriers in materials, including hole transport materials used in organic electronic devices is polarizability.^52,53^ In the context of hole transport materials, polarizability can affect the mobility of charge carriers, including holes, within the material. Materials with high polarizability tend to have a higher interaction with electric fields, which can enhance the mobility of holes and improve the performance of devices such as organic solar cells. As seen in Table 1, the polarizability of GQD increases by replacing three edge hydrogens with selected substituents. Among these substituents, the highest polarizability is observed when hydrogens are replaced with the –S(CH_3_) substituent.

### Frontier orbital levels

**Fig. 2:**
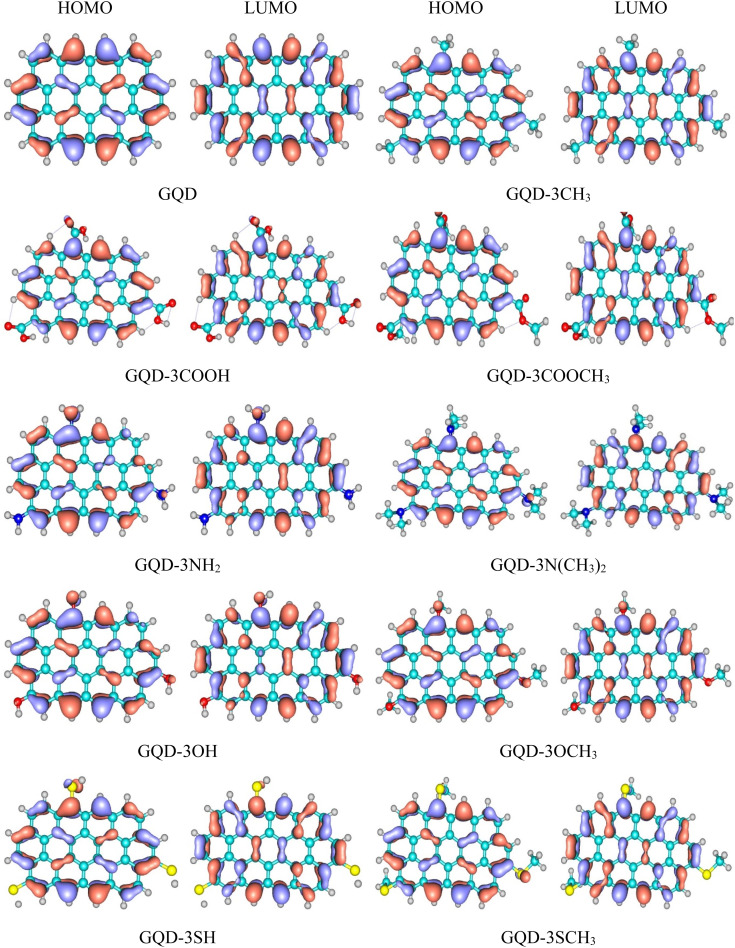
The HOMO and LUMO schema of the GQD and GQD-3Xs.

The transportation of holes through a HTM is intricately connected to the energy levels of its HOMO and LUMO orbitals, which exert a profound influence on the overall efficacy of a PSC. The schemas of HOMO and LUMO of the considered GQD and GQD-3Xs in dichloromethane solvent have been illustrated in Fig. 2. Also, Table 2 reports the energies of HOMO and LUMO of the considered GQD and GQD-3Xs. To be suitable for PSC applications, a molecule must possess a HOMO level that is higher than that of the valence band (VB) the perovskite. MAPbI_3_ (methylammonium lead iodide) is a perovskite material that has been widely studied for its potential use in solar cells. The PSCs based on MAPbI_3_ have shown high power conversion efficiency (PCE), which is a measure of how effectively the solar cell can convert light into electricity.^54–56^ The VB of MAPbI_3_ was measured to be −5.43 eV.^57^ As reported in Table 2, the HOMO energies of all GQD and GQD-3Xs are higher than HOMO level of MAPbI_3_, indicating GQD and GQD-3Xs may be appropriate HTMs for MAPbI_3_ based solar cells.

**Table 2:** Calculated HOMO, LUMO, HOMO–LUMO gap (*E*_g_), and Fermi energy (*E*_f_) of the GQD and GQD-3Xs

	HOMO (eV)	LUMO (eV)	*E*_g_ (eV)	*E*_f_ (eV)
GQD	−4.96	−2.81	2.15	−3.88
GQD-3CH_3_	−4.88	−2.76	2.11	−3.82
GQD-3COOH	−5.30	−3.21	2.09	−4.25
GQD-3COOCH_3_	−5.23	−3.13	2.10	−4.18
GQD-3NH_2_	−4.55	−2.54	2.01	−3.55
GQD-3N(CH_3_)_2_	−4.82	−2.74	2.09	−3.78
GQD-3OH	−4.80	−2.71	2.09	−3.76
GQD-3OCH_3_	−4.86	−2.79	2.07	−3.82
GQD-3SH	−5.02	−2.96	2.06	−3.99
GQD-3SCH_3_	−5.00	−2.94	2.06	−3.97

Another critical requirement for HTMs in PSC applications is their capacity to transport holes to the Au cathode. To fulfill this criterion, the HOMO level of the HTM should be situated below the Fermi level of the Au cathode, which is −5.1 eV.^58^ As seen in Table 2, the HOMO energy levels of GQD-3COOH and GQD-3COOCH_3_ are less than the Fermi level of Au cathode. An important observation to make is that, GQD-3COOH and GQD-3COOCH_3_ possess HOMO energies that are more profound than Spiro-OMeTAD's HOMO energy of −5.20 eV.^59^ As a result, it is plausible to anticipate that GQD-3COOH and GQD-3COOCH_3_ are more suitable for use as HTMs compared to Spiro-OMeTAD.

In addition to proper position of HOMO, the LUMO level of a suitable HTM need to be located higher than the conduction band of MAPbI_3_ perovskite (−3.93 eV).^57^ Preventing the flow of photo-generated electrons from the MAPbI_3_ layer back towards the Au electrode is the key reason for this requirement.^47^ As observed in Table 1, the LUMO levels of all considered GQD and GQD-3Xs are positioned upper than the conduction band of MAPbI_3_ perovskite.

### UV-spectra and exciton binding energy

**Fig. 3:**
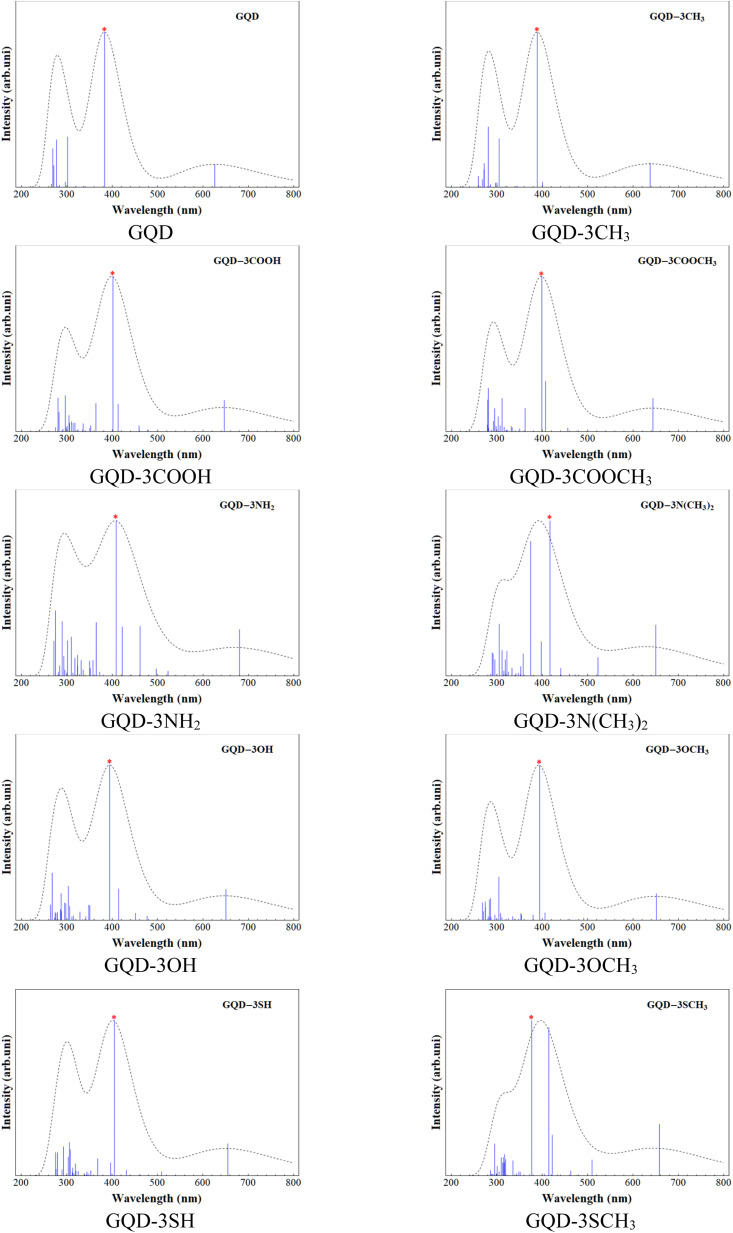
The UV-visible spectra of the GQD and GQD-3Xs.

The UV-visible spectra for the GQD and GQD-3Xs were obtained using TD-DFT at the mentioned level. Table 3 provides the electronic configuration of the first and most intense absorption lines, while Fig. 3 displays the calculated UV-visible spectra with a half width at half height (HWHH) of 0.33 eV. The UV spectra of GQD and GQD-3Xs exhibit three significant peaks: a short peak in the 600–650 nm range, and two intense peaks in the 350–450 nm and 250–350 nm ranges. The short peaks are attributed to the S0 → S1 electron excitation, which corresponds to electron excitation from HOMO to LUMO. The most intense absorption lines in the UV-visible spectra of GQD and GQD-3Xs, except for GQD-3NH_2_, GQD-3N(CH_3_)_2_, GQD-3OH, and GQD-3SCH_3_ are attributed to S0 → S6 electron excitation. S0 → S7 electron excitation accounts for the most intense absorption lines in GQD-3NH_2_, GQD-3N(CH_3_)_2_, and GQD-3OH and S0 → S9 corresponds to the most intensive absorption line in GDQ-3SCH_3_. Furthermore, the light harvesting efficiencies (LHEs) of the GQD and GQD-3Xs using eqn (1) presented in Table 4 demonstrate their suitable light absorption capabilities. As seen, the most LHEs correspond to GQD and GQD-CH_3_ with value of 0.99 while GQD-NH_2_ and GQD-3N(CH_3_)_2_ have the lest LHE with value of 0.87.

**Table 3:** The main electronic configuration (MEC), wavelength (*λ*), oscillator strength (*f*), and the possibility of electronic configuration (*P*) of the first and the most intensive absorption lines of the GQD and GQD-3Xs. The H is HOMO and L is LUMO

Electronic excitation	*λ* (nm)	*f*	MEC	*P*
**GQD**				
S0 → S1	625.88	0.30	H → L	1.00
S0 → S6	383.11	1.99	H → L+2	0.51
			H−1 → L	0.47

**GQD-3CH**_**3**_				
S0 → S1	637.48	0.31	H → L	1.00
S0 → S6	388.96	1.96	H → L+1	0.38
			H−2 → L	0.24
			H−1 → L	0.21
			H → L+2	0.12

**GQD-3COOH**				
S0 → S1	646.75	0.29	H → L	1.00
S0 → S6	401.43	1.44	H → L+3	0.23
			H → L+2	0.21
			H−2 → L	0.21
			H → L+1	0.18
			H−1 → L	0.14

**GQD-3COOCH**_**3**_				
S0 → S1	643.75	0.30	H → L	1.00
S0 → S6	398.75	1.40	H → L+3	0.27
			H−2 → L	0.23
			H → L+1	0.21
			H → L+2	0.13

**GQD-3NH**_**2**_				
S0 → S1	680.33	0.27	H → L	1.00
S0 → S7	408.32	0.89	H → L+3	0.40
			H−3 → L	0.29
			H → L+2	0.11

**GQD-3N(CH**_**3**_ **)**_**2**_				
S0 → S1	649.89	0.29	H → L	1.00
S0 → S7	416.82	0.89	H → L+2	0.26
			H−5 → L	0.22
			H → L+1	0.16
			H−3 → L	0.14

**GQD-3OH**				
S0 → S1	650.45	0.27	H → L	1.00
S0 → S7	394.31	1.36	H → L+3	0.21
			H → L+2	0.18
			H−1 → L	0.17
			H−2 → L	0.15
			H−3 → L	0.13
			H → L+1	0.13

**GQD-3OCH**_**3**_				
S0 → S1	651.33	0.30	H → L	1.00
S0 → S6	394.20	1.75	H → L+2	0.43
			H−1 → L	0.21
			H−2 → L	0.20

**GQD-3SH**				
S0 → S1	655.08	0.32	H → L	1.00
S0 → S6	405.18	1.54	H → L+1	0.39
			H−2 → L	0.19
			H−3 → L	0.15
			H−1 → L	0.11

**GQD-3SCH**_**3**_				
S0 → S1	658.10	0.30	H → L	1.00
S0 → S9	376.76	0.91	H−5 → L	0.68
			H → L+2	0.12

**Table 4:** HOMO–LUMO bandgap energy (*E*_g_), optical bandgap energy (*E*_opt_), oscillator strength of the most intensive absorption line (*f*_max_), exciton binding energy (*E*_b_), and light harvest efficiency (LHE) of the GQD and GQD-3Xs

	*E*_g_ (eV)	*E*_opt_ (eV)	*f*_max_	*E*_b_ (eV)	LHE
GQD	2.15	1.98	1.99	0.17	0.99
GQD-3CH_3_	2.11	1.95	1.96	0.16	0.99
GQD-3COOH	2.09	1.92	1.44	0.17	0.96
GQD-3COOCH_3_	2.10	1.93	1.40	0.17	0.96
GQD-3NH_2_	2.01	1.82	0.89	0.19	0.87
GQD-3N(CH_3_)_2_	2.09	1.91	0.89	0.18	0.87
GQD-3OH	2.09	1.91	1.36	0.18	0.96
GQD-3OCH_3_	2.07	1.90	1.75	0.17	0.98
GQD-3SH	2.06	1.89	1.54	0.17	0.97
GQD-3SCH_3_	2.06	1.88	0.91	0.18	0.88

The required energy for S0 → S1 excitation corresponds to optical bandgap (*E*_opt_). As shown in eqn (2), the difference between *E*_g_ and *E*_opt_ is the exciton binding energy (*E*_b_) which indicates the strength of the coulombic attraction between the excited electron and the hole.^46^ A large exciton binding energy implies a strong electron–hole interaction, which can result in reduced charge separation efficiency and decreased device performance, particularly for solar cells, where efficient charge separation is critical for high power conversion efficiency. Conversely, a small exciton binding energy can indicate weak electron–hole interactions, facilitating efficient charge separation and transport, making the material more suitable for optoelectronic applications. The *E*_b_ of the GQD and GQD-3Xs were found to be within the range of 0.17 to 0.19 eV (Table 4), indicating weak electron–hole interactions and high charge transfer in these materials.

### Hole reorganization energy and hole mobility

The hole reorganization energy (*λ*_h_) is the energy change that occurs in a system when it undergoes structural relaxation following the loss of electrons.^60^*λ*_h_ is calculated by:

3 *λ*_h_ = (*E*^+^_0_ − *E*_+_) + (*E*^0^_+_ − *E*_0_)

*E*^+^_0_ and *E*_+_ are the cationic energies of optimized neutral and cationic structures, respectively. Also, *E*^0^_+_ and *E*_0_ denote the neutral energies of optimized cationic and neutral structures. Minimizing the *λ*_h_ is crucial for achieving high charge carrier mobility in organic semiconductors.^60^Table 5 presents a comparison of the *λ*_h_ values for GQD and GQD-3Xs. The table indicates that, with the exception of GQD-3N(CH_3_)_2_ and GQD-3NH_2_, the *λ*_h_ values of GQD and GQD-3Xs are all less than *λ*_h_ values of Spiro-OMeTAD (163.31 meV (ref. 8, 47, 60 and 61)), suggesting that they may have a better mobility than Spiro-OMeTAD.

**Table 5:** Hole reorganization energy (*λ*_h_), hole hopping rate (*k*_h_), transfer integral (*ν*_h_), centroid to centroid distance (*r*), hole mobility (*μ*), open circuit voltage (*V*_OC_), and fill factor (FF) of the GQD and GQD-3Xs

	*λ*_h_ (meV)	*k*_h_ (s^−1^)	*ν*_h_ (eV)	*r*	*μ* (cm^2^ V^−1^ s^−1^)	*V*_OC_ (V)	FF
GQD	72.95	2.19 × 10^14^	0.085	4.15	2.45	0.73	0.85
GQD-3CH_3_	77.13	1.17 × 10^12^	0.0064	7.89	0.047	0.65	0.84
GQD-3COOH	100.04	1.15 × 10^14^	0.076	3.45	0.89	1.07	0.89
GQD-3COOCH_3_	91.30	4.66 × 10^12^	0.014	6.67	0.13	1.00	0.88
GQD-3NH_2_	196.80	9.17 × 10^12^	0.041	5.85	0.20	0.32	0.74
GQD-3N(CH_3_)_2_	317.26	1.54 × 10^13^	0.11	3.43	0.12	0.59	0.83
GQD-3OH	113.30	7.46 × 10^12^	0.021	7.67	0.28	0.57	0.82
GQD-3OCH_3_	127.64	8.79 × 10^12^	0.025	10.40	0.62	0.63	0.83
GQD-3SH	112.13	3.19 × 10^11^	0.0044	9.89	0.020	0.79	0.86
GQD-3SCH_3_	79.95	1.31 × 10^13^	0.022	11.40	1.10	0.77	0.86

The most stable molecular structure pairs of GQD and GQD-3Xs, and the Einstein ration were used for modeling their hole mobility:^62^4
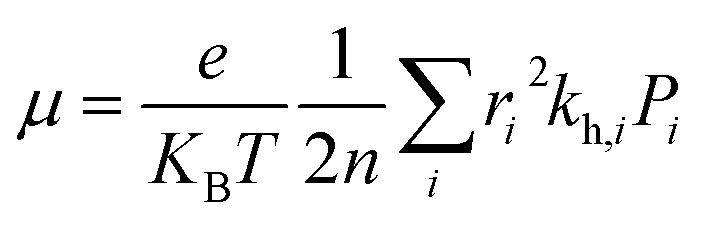
where *e*, *K*_B_, and *T* indicate unit electronic charge, Boltzmann constant (8.61733034), and temperature (298 K), respectively. *r*_*i*_ is centroid to centroid distance. Also, *k*_h,*i*_ and *P*_*i*_ are the hole hopping rate and hoping possibility, respectively, and are obtained employing following equations:^62^5
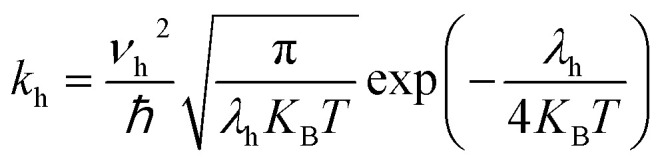
6
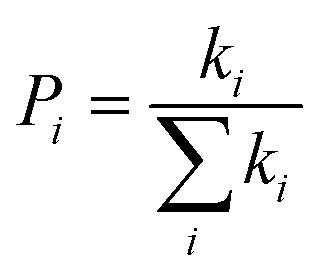


In eqn (5), *ν*_h_ is transfer integral and proximately obtained by:^8^7
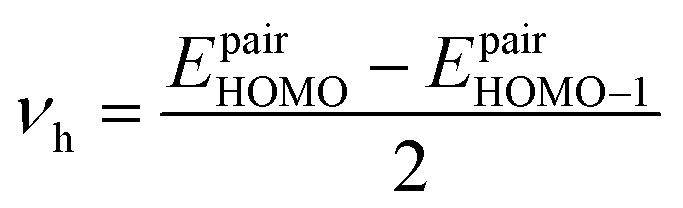


The hole mobility along with hole hopping rate, transfer integral, and centroid to centroid distance of the considered GQD and GQD-3Xs have been listed in Table 5. As seen, the hole mobility of all considered GQD and GQD-3Xs is more than calculated and experimental hole mobility values of Spiro-OMeTAD (2 × 10^−4^ to 6 × 10^−3^ cm^2^ V^−1^ s^−1^).^51,62,63^ Among them, GQD, GQD-3SCH_3_, and GQD-3COOH have the most hole mobility with values of 2.45, 1.10, and 0.89 cm^2^ V^−1^ s^−1^, respectively.

### Performances of PSCs

The performance of PSCs are proportional to their open circuit voltage (*V*_OC_) and fill factor (FF) as described in following equation:^64^

8 PC ∝ *V*_OC_FF

*V*_OC_ and FF can be calculated by eqn (6) and (7):^65^

9 *V*_OC_ = (1/*e*)(*E*_LUMO_(acceptor) − *E*_HOMO_(donor)) − 0.3 V

In this case, MAPbI_3_ perovskite is considered as acceptor and the GQD and GQD-3Xs are donors.10
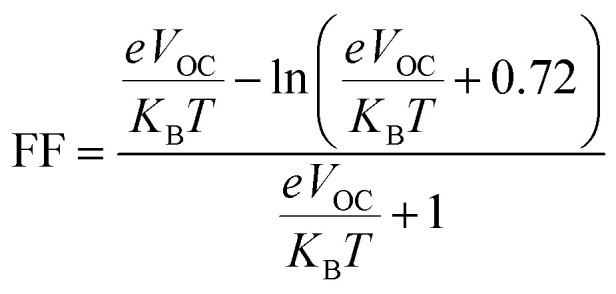


Table 5 have been also reported *V*_OC_ and FF of the GQD and GQD-3Xs. As seen, the most values of *V*_OC_ are relative to GQD-3COOH, GQD-3COOCH_3_, GQD-3SH, and GQD-3SCH_3_ with values of 1.07, 1.00, 0.79, and 0.77 eV, respectively.

Table 6 presents a summary of the results obtained from evaluating various criteria for HTMs in the case of GQD and GQD-3Xs, and compares them to Spiro-OMeTAD. It is evident from the table that only GQD-3COOH and GQD-3COOCH_3_ meet all the necessary criteria to be considered suitable HTMs. Additionally, these GQD-3Xs exhibit higher hole mobility and open circuit voltage compared to Spiro-OMeTAD. Consequently, GQD-3COOH and GQD-3COOCH_3_ can be effectively utilized as efficient HTMs for perovskite solar cells.

**Table 6:** A summary for the comparison of GQD and GQD-3Xs in various criteria of HTMs

	H1^a^^,^^d^	H2^b^^,^^d^	L1^c^^,^^d^	*D* (gas)	*E*_b_	LHE	*μ*	*V*_OC_	FF
GQD	0.47	−0.14	1.12	0.00	0.17	0.99	2.45	0.73	0.85
GQD-3CH_3_	0.55	−0.22	1.17	0.13	0.16	0.99	0.047	0.65	0.84
GQD-3COOH	0.13	0.20	0.72	6.20	0.17	0.96	0.89	1.07	0.89
GQD-3COOCH_3_	0.20	0.13	0.80	6.78	0.17	0.96	0.13	1.00	0.88
GQD-3NH_2_	0.88	−0.55	1.39	3.46	0.19	0.87	0.20	0.32	0.74
GQD-3N(CH_3_)_2_	0.61	−0.28	1.19	1.26	0.18	0.87	0.12	0.59	0.83
GQD-3OH	0.63	−0.30	1.22	1.67	0.18	0.96	0.28	0.57	0.82
GQD-3OCH_3_	0.57	−0.24	1.14		0.17	0.98	0.62	0.63	0.83
GQD-3SH	0.41	−0.08	0.97	1.78	0.17	0.97	0.020	0.79	0.86
GQD-3SCH_3_	0.43	−0.10	0.99	3.43	0.18	0.88	1.10	0.77	0.86
Spiro-OMeTAD	0.23	0.10	1.43	—	—	—	0.0057 (ref. 35)	0.97^e^	0.88^e^

a H1 = *E*_HOMO_(HTM) − *E*_HOMO_(perovskite).

b H2 = *E*_Fermi_(Au) − *E*_HOMO_(HTM).

c L1 = *E*_LUMO_(HTM) − *E*_LUMO_(perovskite).

d Negative sign indicates that the compound is not a suitable HTM.

e Calculated by eqn (9) and (10).

## Conclusion

In this study, the suitability of graphene quantum dot (GQD) and certain edge functionalized GQDs (GQD-3Xs, where X = CH_3_, COOH, COOCH_3_, NH_2_, NMe_2_, OH, OMe, SH, and SMe) as HTMs in PSCs was studied using DFT and TD-DFT. Specific criteria must be met for a compound to be considered an appropriate HTM in PSC, including the positioning of the HOMO of the HTM higher than the valence band (VB) of the perovskite, equal to or lower than the Fermi level of the gold cathode, and the LUMO energy level of the HTM higher than the conduction band (CB) of the perovskite. For this study, MAPbI_3_ perovskite, which is widely used in PSCs, with VB and CB energy levels of −5.43 and −3.93 eV, respectively, was selected. Among the GQD and GQD-3Xs considered, the DFT calculations clarified that the HOMO levels of GQD and GQD-3Xs except for GQD-3COOH and GQD-3COOCH_3_ were located higher than the Fermi level of Au cathode. As a result, these compounds do not meet the criteria, and they are not suitable as HTMs in PSC.

Solubility is a critical characteristic of HTMs in the development of organic electronic devices. The solubility of GQD and GQD-3Xs in dichloromethane was investigated in this study. It was found that GQD-COOH and GQD-COOCH_3_ had the highest solubility, but these compounds did not meet the criteria to be used as suitable HTMs in PSCs. Among the compounds that met the criteria, GQD-3OH and GQD-3OCH_3_ had the best solubility. The LHE and *E*_b_ of GQD and GQD-3Xs were determined by analyzing their UV-visible spectra. Among the compounds that met the criteria, their LHE was found to be greater than 0.87, indicating their potential for effective light harvesting. The calculated *E*_b_ values fell within the range of 0.17 to 0.19, suggesting their potential for efficient charge separation. The investigation of the hole reorganization energies (*λ*_h_) and the hole mobility of GQD and GQD-3Xs revealed that the hole mobility of all compounds considered may be higher than Spiro-OMeTAD and other benzene derivatives commonly used as HTMs in PSCs. Additionally, the *V*_OC_ and fill factor (FF) of the GQD and GQD-3Xs that can be used as HTMs fall within the ranges of 0.57 to 1.07 V and 0.74 to 0.89, respectively, indicating their appropriate HTM performance in MAPbI_3_ PSCs. Overall, due to its high solubility, small *λ*_h_, and appropriate performance, GQD-COOH and GQD-COOCH_3_ were found to be the most suitable HTMs among the compounds considered in this study.

## Conflicts of interest

The authors declare that they have no conflict of interest.

## Supplementary Material
